# Cisplatin Induces Pyroptosis via Activation of MEG3/NLRP3/caspase-1/GSDMD Pathway in Triple-Negative Breast Cancer

**DOI:** 10.7150/ijbs.60292

**Published:** 2021-06-22

**Authors:** Honglin Yan, Bin Luo, Xiaoyan Wu, Feng Guan, Xinxin Yu, Lina Zhao, Xiaokang Ke, Juan Wu, Jingping Yuan

**Affiliations:** Department of Pathology, Renmin Hospital of Wuhan University, Wuhan, Hubei 430060, P.R. China.

**Keywords:** triple-negative breast cancer, pyroptosis, cisplatin, MEG3, chemotherapy

## Abstract

Cisplatin (DDP) was reported to improve pathological complete response (pCR) rates in triple-negative breast cancer (TNBC) patients, however, the molecular mechanism still remains largely unknown. Emerging evidence suggested that some chemotherapeutic drugs played anti-tumor effects by inducing cell pyroptosis. Nevertheless, whether pyroptosis contributes to the DDP-induced anti-tumor effect in TNBC remains unexploited. In the present study, NLRP3/caspase-1/GSDMD pyroptosis pathway was involved in the DDP-induced anti-tumor effect of TNBC *in vitro* and *in vivo*, providing evidence that DDP might induce pyroptosis in TNBC. Moreover, DDP activated NLRP3/caspase-1/GSDMD pyroptosis pathway by up-regulating the long non-coding RNA (lncRNA) maternally expressed gene 3 (MEG3). Furthermore, knockdown of MEG3 not only partly abolished the activation effect of DDP on NLRP3/caspase-1/GSDMD pathway-mediated pyroptosis, but also reversed the suppression of DDP on tumor growth and metastasis ability *in vitro* and *in vivo,* further confirming that MEG3 may partially mediate the pyroptotic signaling upon DDP treatment. Thus, our data uncovered a novel mechanism that DDP induced pyroptosis via activation of MEG3/NLRP3/caspase-1/GSDMD pathway in TNBC to exert anti-tumor effects, which may help to develop new strategies for the therapeutic interventions in TNBC.

## Introduction

Triple-negative breast cancer (TNBC) is a subtype of breast cancer, which is commonly defined as the absence of estrogen receptor (ER), progesterone receptor (PR), and human epidermal growth factor receptor 2 (HER2). TNBC accounts for 10 to 17% of all breast carcinomas, which is more aggressive and has higher recurrence and mortality than other subtypes 1. Since TNBC patients cannot benefit from endocrine therapy or HER2-targeted agents, chemotherapy is still the most important means of systemic treatment for TNBC patients 2, 3. Neoadjuvant chemotherapy (NACT) for breast cancer is a new strategy before surgical extraction of tumors with the aim of rendering an otherwise inoperable tumor operable, allowing conservative surgery, improving long-term outcomes for the patients, and providing the opportunity to test new drugs preoperatively 4. Recently, the addition of cisplatin (DDP) or other platinum drugs to NACT has been demonstrated to improve pathological complete response (pCR) rates in TNBC patients 5-7. However, the molecular mechanism still remains largely unknown.

It is widely known that chemotherapeutic drugs exert anti-tumor effects by inhibiting cell proliferation and inducing programmed cell death (PCD). Traditionally, apoptosis was considered to be the main way of PCD induced by chemotherapeutic drugs, however, tumor cells can escape apoptosis through various mechanisms, resulting in natural or secondary resistance to chemotherapy 8. Other forms of PCD may be a way to overcome chemotherapy resistance. Recent studies have shown that several chemotherapeutic drugs could also play anti-tumor effects by inducing cell pyroptosis, which is an emerging type of programmed cell death (PCD) 9-11. Unlike apoptosis, pyroptosis is a pro-inflammatory PCD characterized by cell swelling, rupture of the plasma membrane, formation of large bubbles on the cell membrane, and the release of pro-inflammatory cytokines 9-12. It has been demonstrated that the activation of NOD-like receptor family pyrin domain-containing 3 (NLRP3) inflammasome is required for pyroptosis in various diseases 13-15. NLRP3 inflammasome is a multimeric protein complex formed by the innate immune sensor protein NLRP3. Upon activation, NLRP3 oligomerizes and binds to adaptor protein ASC, then recruits caspase-1 to form NLRP3 inflammasome 16. The assembly of NLRP3 inflammasome leads to the cleavage and activation of caspase-1, which further cleaves gasdermin D (GSDMD) and releases the cleaved GSDMD N-terminal fragment (GSDMD-N) to form membrane pore, thereby promoting pyroptosis 17-19. On the other hand, activated caspase-1 promotes the maturation and secretion of pro-inflammatory cytokines, such as IL-1β and IL-18 20, 21. However, the role of pyroptosis in the anti-tumor effect of DDP in TNBC, as well as the underlying mechanism remain poorly investigated.

Long non-coding RNAs (lncRNAs) are a type of non-coding RNAs that are longer than 200 bps in length and have little or no protein-coding capacity 22. Recently, a growing body of evidence uncovered that lncRNAs are emerging regulators of cell pyroptosis 23-26. Maternally expressed gene 3 (MEG3) is an imprinted gene in tumorigenesis and functions as a lncRNA. A most recent study indicated that MEG3 promoted pyroptosis by targeting NLRP3 inflammasome 24. However, whether MEG3/NLRP3 axis induces pyroptosis of TNBC and whether it mediates the anti-tumor effect of DDP in TNBC has not been reported yet.

Here, we found that NLRP3/caspase-1/GSDMD pathway-mediated pyroptosis was activated in response to DDP treatment *in vitro* and *in vivo*. Notably, DDP activated NLRP3/caspase-1/GSDMD pyroptosis pathway by up-regulating MEG3. Knockdown of MEG3 abolished the activation effect of DDP on NLRP3/caspase-1/GSDMD pathway-mediated pyroptosis, and reversed the suppression of DDP on tumor growth and metastasis ability *in vitro* and *in vivo*. We demonstrated for the first time that DDP induced pyroptosis via activation of MEG3/NLRP3/caspase-1/GSDMD pathway in TNBC, which may help to develop new strategies for the therapeutic interventions in TNBC.

## Materials and methods

### Patients and specimens

The twenty patients included in this study were selected from the TNBC patients who underwent DDP-based NACT at Renmin Hospital of Wuhan University between January 2017 and September 2019. All patients were pathologically diagnosed with invasive TNBC via core needle biopsy. Formalin-fixed and paraffin-embedded tissues of fine-needle aspiration biopsy (pre-NACT) and resected specimens (post-NACT) were collected. Prior information and written consent were obtained from all participants before starting our project. This study was approved by the Ethics Committee of Renmin Hospital of Wuhan University.

### H&E and immunohistochemical staining

Tissue samples isolated from TNBC patients or xenograft tumors were fixed with formalin and embedded in paraffin. Then the tissue samples were cut at 4 μm, stained with H&E. Immunohistochemical staining was performed using a standard Envision complex method. The primary antibodies used were as follows: anti-GSDMD (Proteintech, Wuhan, China, 20770-1-AP), anti-NLRP3 (Abcam, Cambridge, MA, UK, ab214185), anti-caspase-1 (Proteintech, 22915-1-AP), anti-IL-18 (Proteintech, 60070-1-Ig), anti-IL-1β (Proteintech, 66737-1-Ig), anti-Ki67 (ready-to-use; DAKO, Glostrup, Denmark, M7240, clone MIB1).

### Histological Scoring

Five randomly selected high-power fields (400×) were averaged in each case to calculate the immunoreactive result. Sections were independently examined and scored by two experienced pathologists who were blinded to the clinical data. The expression of GSDMD, NLRP3, caspase-1, IL-18, and IL-1β were scored by multiplying the mean signal intensity (on a scale of 0-3: 0, no staining; 1, light staining; 2, moderate staining; and 3, strong staining) and the proportion of positive tumor cells (on a scale of 0-4: 0, 0%; 1, 1-25%; 2, 26-50%; 3, 51-75%; and 4, 76-100%). The final immunoreactive score was the mean of scores from the three investigators. Ki67 was scored as the percentage of positive tumor cells nuclei by counting a range of 400-500 cells (depending on the cellularity of the specimen), including hot spot areas.

### Cell culture

The MDA-MB-231 cell line was maintained in our laboratory and cultured in Dulbecco's modified Eagle's minimal essential medium (DMEM; Gibco; Thermo Fisher Scientific, Inc., Waltham, MA, USA) supplemented with 10% fetal bovine serum (FBS) and 1% penicillin-streptomycin in an incubator that contained 5% CO_2_ at 37 °C.

### 3-(4,5-dimethyl-2-thiazolyl)-2, 5-diphenyltetrazolium bromide (MTT) assay

MDA-MB-231 cells were seeded into 96-well plates at a density of 7000 cells/well. To measure the cell viability, 20 μl MTT (Sigma-Aldrich, St Louis, MO, USA) was added at 5 mg/mL to each well and incubated for 4 h, and the solubilized formazan was resolved by DMSO. The absorbance of each well was measured at 490 nm by PerkinElmer Victor3 1420 Multilabel Counter (Waltham, MA).

### Flow cytometry analysis

MDA-MB-231 cells were harvested, washed with PBS, and stained with the FITC-labeled Annexin V and propidium iodide (PI) for 15 min at room temperature in the dark using the Annexin V-FITC/PI Apoptosis Detection Kit (Vazyme Biotech, Tianjin, China). Then 150 μl 1 × binding buffer was added to each sample. Data was obtained using CytoFLEX (Beckman Coulter Inc, Brea, CA, USA) and analyzed by CytExpert software.

### Lactate dehydrogenase (LDH) release assay

Culture supernatants were harvested and centrifuged at 300×g for 10 min. 20 μl supernatants were collected, and LDH release was measured using the LDH assay kit (Nanjing Jiancheng Bioengineering Institute, Nanjing, China) according to the manufacturer's instruction. The absorbance value was measured at 450 nm.

### Western blot analysis

Total proteins were extracted with RIPA lysis buffer (Sigma-Aldrich) on ice, and western blot analysis was performed as described previously 27. The primary antibody information was as follows: anti-cleaved N-terminal GSDMD (Abcam, ab215203), anti-NLRP3 (Abcam, ab214185), anti-caspase-1 (Proteintech, 22915-1-AP), anti-IL-18 (Proteintech, 60070-1-Ig), anti-IL-1β (Proteintech, 66737-1-Ig), and anti-GAPDH (Abcam, ab37168). Signals were visualized using Odyssey infrared imaging system (LI-COR, Lincoln, NE, USA).

### ELISA

The protein concentration of IL-18 and IL-1β was measured in culture medium supernatant of MDA-MB-231 cells using the human IL-18 ELISA kit (Proteintech) and the human IL-1β ELISA kit (Proteintech). ELISA assays were performed according to the manufacturer's instructions. The quantity of protein is measurable at 450 nm.

### RNA isolation and quantitative reverse transcription polymerase chain reaction (qRT-PCR)

Total RNA was isolated from cells or tissues by using Trizol reagents (TaKaRa, Tokyo, Japan). cDNA was synthesized by using M-MLV reverse transcriptase (Invitrogen) and reverse transcription primers Oligo(dT). PCR amplification was performed with SYBR Green Real-Time PCR Master Mixes (Thermo Fisher, Waltham, MA, USA) following the manufacturers' instructions on a 7900HT Fast Real-Time PCR machine (Applied Biosystems). The relative expression level of genes was normalized to the internal reference gene GAPDH. The primers sequence were used as follows: MEG3 forward, 5'-GAGAAAATGCAGGCCGAGAG-3'; MEG3 reverse, 5'-CCCCATTACTGTCCCCAAGT-3'; GAPDH forward, 5'-TGTTTCCTCGTCCCGTAGA-3'; GAPDH reverse, 5'-GATGGCAACAATCTCCACTTTG-3'.

### MEG3 knockdown *in vitro* and* in vivo*

To inhibit the MEG3 expression in MDA-MB-231 cells, three siRNAs targeting MEG3 (si-MEG3) and scrambled control (si-s-MEG3) were designed. MDA-MB-231 cells were transfected with siRNAs by using Lipofectamine 2000 (Invitrogen, CA, USA) according to the manufacturer's instructions. The sequences for si-MEG3 were as follows: si-MEG3-1 sense, CCCUCUUGCUUGUCUUACUTT; si-MEG3-1 antisense, AGUAAGACA AGCAAGAGGGTT; si-MEG3-2 sense, GCUCAUACU UUGACUCUAUTT; si-MEG3-2 antisense, AUAGAGUCAAAGUAUGAGCTT; si-MEG3-3 sense, GAUCCCACCAACAUACAAATT; si-MEG3-3 antisense, UUUGUAUGUUGGUGGGAUCTT. All of the three siRNAs targeting MEG3 had significant inhibitory effects on MEG3, especially si-MEG3-2 had the largest inhibitory effect on MEG3 compared to the si-s-MEG3 control. Thus, si-MEG3-2 was chosen in all subsequent *in vitro* experiments.

In order to establish a MEG3 knockdown xenograft model, a stable MEG3 knockdown cell line was first constructed. The shRNA direct against the MEG3 gene was designed and constructed into the lentivirus expressing vector pLKO.1 GFP shRNA, and was referred as to Lv-sh-MEG3. The plasmid carrying a non-targeting sequence was used as a negative control (NC) of sh-MEG3 and was referred as to Lv-sh-NC. The sequences for MEG3 shRNAs were as follows: 5'-CCGGGCTCATACTTTGACTCTATTTCTCGAGAAATAGAGTCAAAGTATGAGCTTTTTG-3'.

### Colony formation assay

MDA-MB-231 cells were trypsinized and seeded at 500 cells per 6cm-dishes with 10 ml of the culture medium. The cells were grown for an additional 2 weeks at 37 °C, 5% CO_2_, and 100% humidity without changing the medium. Then, colonies were washed with PBS, fixed with 4% paraformaldehyde, and stained with 0.05% crystal violet for 5 min. Image J software was used to quantify the number of colonies.

### Transwell assay

Transwell chambers (Corning Inc., Corning, NY, USA) were used to evaluate the migration ability of MDA-MB-231 cells. Cells were resuspended by trypsin to a density of 1.5×10^5^ cells/ml. 200 µl cell suspension was seeded in the upper chamber. And 600 µl DMEM supplemented with 10% fetal bovine serum was added to the lower chamber. After incubation for 24 h, migrated cells were fixed with 4% paraformaldehyde at room temperature for 15 min. After that, cells were stained with 0.1% crystal violet for 15 min and stained cells were quantified under a microscope (DM1000; Leica Microsystems GmbH, Wetzlar, Germany).

### Wound healing test

MDA-MB-231 cells were seeded into 6-well plates at a density of 1×10^5^ cells/well. After 24 h, a sterile pipette tip was used to create streaks in the cell monolayers. Then the cells were washed with PBS and incubated with fresh serum-free DMEM in the incubator at 37°C. Images of the wound area were taken at four different time points (0, 12, 24, and 48 h).

### *In vivo* mice xenograft assay

The female BALB/c nude mice (N = 24, age 6-8 weeks) were purchased from the Beijing HFK Bioscience Co., Ltd (Beijing, China). All of the research protocols were approved by the committee on animal experimentation of the Institutional Animal Care and Ethical Standards of Renmin Hospital of Wuhan University. Logarithmic growth phase of the MDA-MB-231 cell line, the stable expressing Lv-sh-NC cell line (Lv-sh-NC) or the stable MEG3-knockdown cell line (Lv-sh-MEG3) were transplanted into the right thoracic mammary fat pads of female BALB/c mice. When the tumor size reached approximately 100 mm^3^ (at about day 7), the mice were equally divided into 3 groups, including PBS negative control group, Lv-sh-NC + DDP treatment group, Lv-sh-MEG3 + DDP treatment group. In the PBS negative control group, the MDA-MB-231 xenograft mice received 200ul/20g PBS by intravenous injection as a control. In the Lv-sh-NC + DDP treatment group, the Lv-sh-NC xenograft mice were injected intraperitoneally with 5 mg/kg DDP twice a week and continued to 24 days. In the Lv-sh-MEG3 + DDP treatment group, Lv-sh-MEG3 xenograft mice were injected intraperitoneally with 5 mg/kg DDP twice a week and continued to 24 days. Day 0 was defined as the day before PBS or DDP injection. Tumor sizes were measured every 4 days, and the tumor volumes were calculated using the following formula: tumor volume = (length × width × width) / 2. At the end of the study, the mice were anesthetized and sacrificed. Tumor tissues and other organs including lung, bone, liver, brain, kidney, and heart were carefully removed for further investigation.

### Statistical analysis

Statistical analysis was performed using GraphPad Prism software. Measurement data were expressed as means ± standard deviation (SD). The statistical significance between two groups and among multiple groups was compared using Student's *t*-test and one-way ANOVA, respectively. The immunoreactivity scoring of pyroptosis-related proteins in pre-NACT and post-NACT from matched breast cancer biopsies were compared using paired-samples *t*-test. *P*<0.05 was considered significant. *P < 0.05, **P < 0.01, ***P < 0.001; ^#^*P* < 0.05, ^##^*P* < 0.01, ^###^*P* < 0.001.

## Results

### Pyroptosis-related proteins are increased post DDP-based neoadjuvant chemotherapy

**Figure 1:**
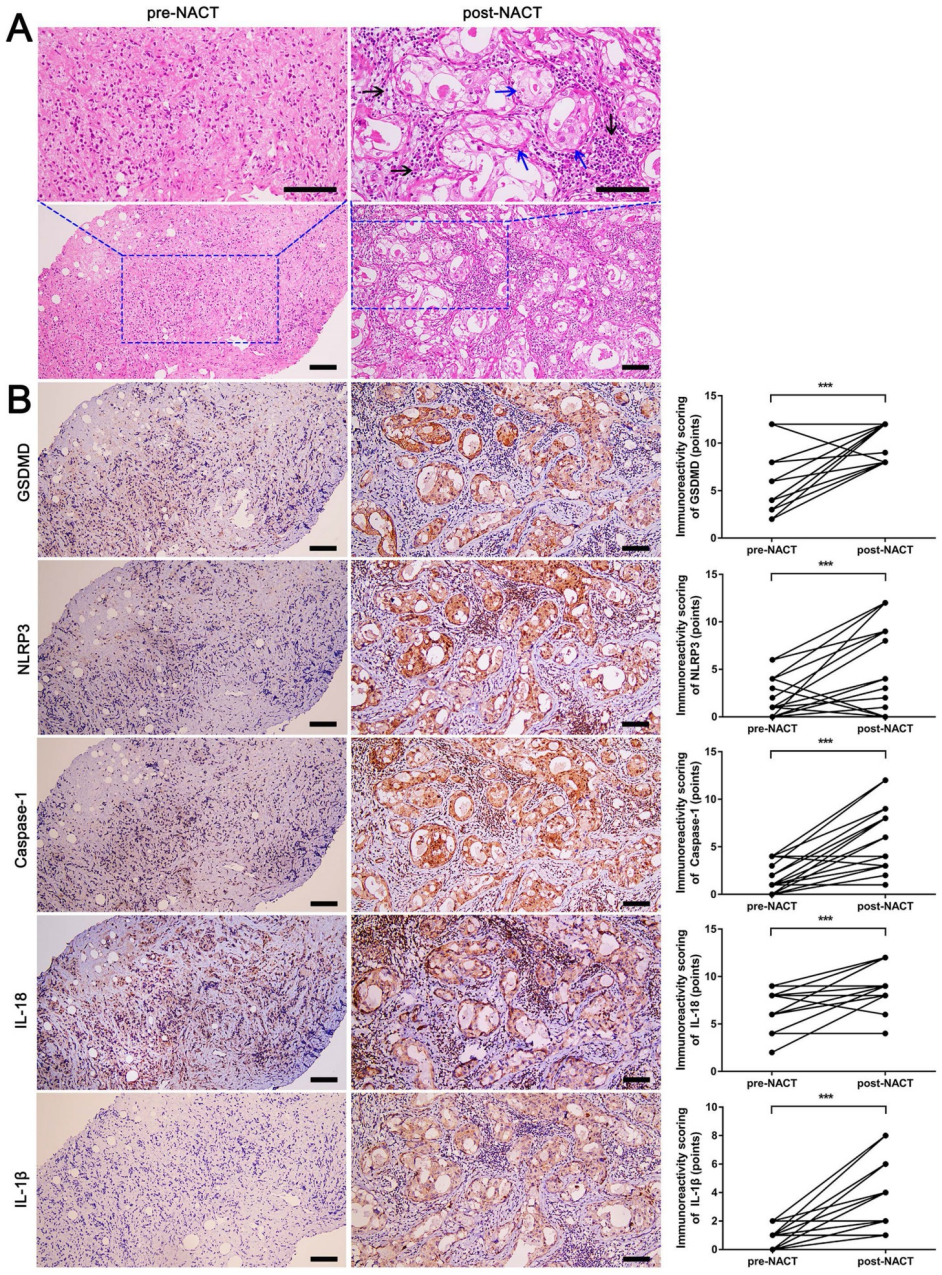
** H&E staining and immunohistochemical staining of pyroptosis-related proteins in fine-needle aspiration biopsy (pre-NACT) and resected specimens (post-NACT) from 20 matched breast cancer biopsies. A)** H&E staining in matched pre- and post-NACT tumor specimens. Blue arrows indicated the tumor cells with pyroptosis-like changes. Black arrows indicated inflammatory cells around the tumor bed. **B)** Immunohistochemical staining of GSDMD, NLRP3, caspase-1, IL-18, and IL-1β expression in matched pre- and post-NACT tumor specimens. Scale bar, 50 µm. ***P < 0.001.

Since NACT can serve as an *in vivo* model to assess the tumor's response to therapy, twenty patients treated with DDP-based NACT were enrolled in our study to evaluate the pathological changes and expression of pyroptosis-related proteins. As shown in Fig. 1A, reactive changes in the breast surgical specimen after chemotherapy included cell swelling, unclear cell membrane boundary, enlarged nuclei, abundant eosinophilic cytoplasm, and cytoplasmic vacuolization (blue arrows). Different numbers of lymphocytes and other inflammatory cells were also observed around the tumor bed (Fig. 1A, black arrows). It is known that pyroptosis is characterized by cell swelling and rupture of the plasma membrane, and is associated with inflammation 9-12. Thus, some tumor morphological characteristics post DDP-based NACT were similar to those of pyroptosis. Moreover, GSDMD, the effector molecule of pyroptosis, was remarkably elevated in post-NACT specimens than in pre-NACT specimens (Fig. 1B). Meanwhile, NLRP3, caspase-1, IL-18, and IL-1β were also upregulated in post-NACT specimens (Fig. 1B). Thus, these findings suggested that DDP-based NACT induced pyroptosis-like changes in TNBC tissues and up-regulated pyroptosis-related proteins.

### DDP activates NLRP3/caspase-1/GSDMD-mediated pyroptosis in MDA-MB-231 cells

**Figure 2:**
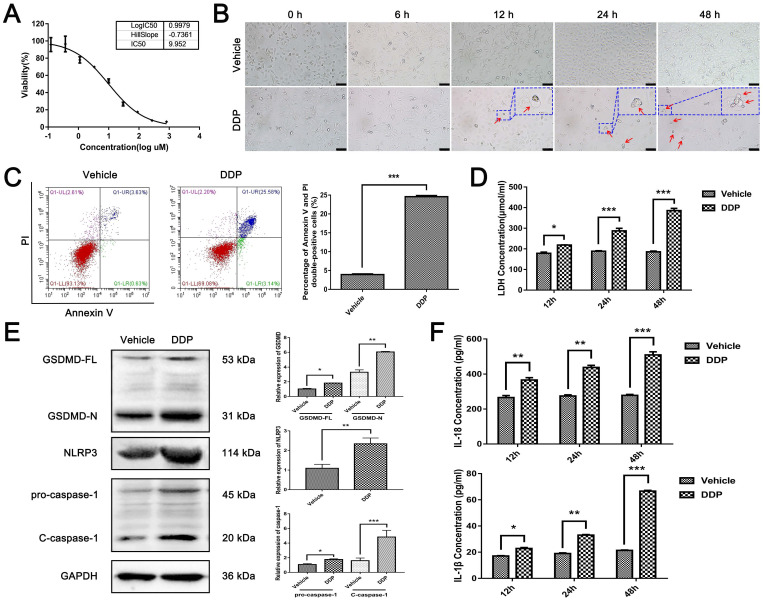
** DDP activated NLRP3/caspase-1/GSDMD-mediated pyroptosis in MDA-MB-231 cells. A)** MDA-MB-231 cells were treated with various concentrations (0, 1/27, 1/9, 1/3, 1, 3, 9, 27, 81, 243 ug/ml) of DDP for 48 h, and cell viability was then determined by the MTT assay. **B)** Representative microscopic images of MDA-MB-231 cells after DDP treatment for the indicated time by living cell imaging. The red arrows indicated pyroptotic cells with characteristic balloons extending from the cellular membrane. The larger images were at the upper right. Scale bar, 50 µm. **C)** The type of cell death after DDP treatment for 24 h in MDA-MB-231 cells was confirmed by flow-cytometry analyses using annexin V and PI staining. **D)** Cytotoxicity of MDA-MB-231 cells after DDP treatment for the indicated time was measured by LDH release in the culture supernatants. **E)** Western blot analysis of pyroptosis-related proteins in MDA-MB-231 cells with or without DDP treatment for 48 h. GAPDH was included as a loading control. **F)** The levels of IL-18 and IL-1β in cell culture supernatant after DDP treatment for the indicated time were determined by ELISA assay. The dose of DDP in the above-mentioned* in vitro* experiments was 9.952 µM. All data are representative of three independent experiments. Data are shown as mean ± SD. *P<0.05; **P<0. 01; ***P<0.001.

Since pyroptosis-related proteins are up-regulated post DDP-based NACT, we next assessed the role of DDP on pyroptosis *in vitro*. Firstly, the median inhibitory concentration (IC50) of DDP on MDA-MB-231 cells was examined by MTT assay. As shown in Fig. 2A, the cell viability of MDA-MB-231 cells responding to DDP treatment decreased significantly in a dose-dependent manner, and the IC50 of DDP was about 9.952 μM (2.986 μg/ml) after 48 h-treatment. Thus, a concentration of 9.952 μM was applied in subsequent experiments. Then, a time-course study of MDA-MB-231 cells treated with DDP was performed to observe the morphology by living cell imaging. Notably, after 12 h of treatment with DDP, the morphology of the cells became swollen with characteristic large bubbles blowing from the plasma membrane (Fig. 2B, red arrows), which exactly resembled the pyroptosis. Since annexin V and PI can stain pyroptotic cells due to the membrane rupture, flow-cytometry analyses using annexin V and PI double staining were performed. As shown in Fig. 2C, the percentage of annexin V and PI double-positive cells was significantly elevated after DDP treatment. Consistent with these results, the increase of the LDH release was observed in a time-dependent manner after DDP treatment (Fig. 2D), indicating that DDP interrupted the cell membrane integrity of MDA-MB-231 cells. Thus, DDP may trigger pyroptosis in MDA-MB-231 cells. Moreover, western blot indicated that not only the full-length GSDMD (GSDMD-FL) but also the N-terminal cleavage product of GSDMD (GSDMD-N) were upregulated in MDA-MB-231 cells exposed to DDP cytotoxicity, indicating that cleaved GSDMD was induced by DDP (Fig. 2E). Additionally, NLRP3, pro-caspase-1, and cleavage of caspase-1 (C-caspase-1) were all elevated after DDP treatment (Fig. 2E), suggesting that NLRP3 inflammasome might be activated. Furthermore, accumulation of IL-18 and IL-1β were also observed in a time-dependent manner in the supernatant of DDP-treated MDA-MB-231 cells culture medium as measured by ELISA, further confirming the pro-inflammatory role of DDP (Fig. 2F). Collectively, these results indicated that DDP activated the NLRP3/caspase-1/GSDMD pathway in MDA-MB-231 cells, which was associated with the occurrence of pyroptosis.

### DDP activates NLRP3/caspase-1/GSDMD pathway by up-regulating MEG3 expression

**Figure 3:**
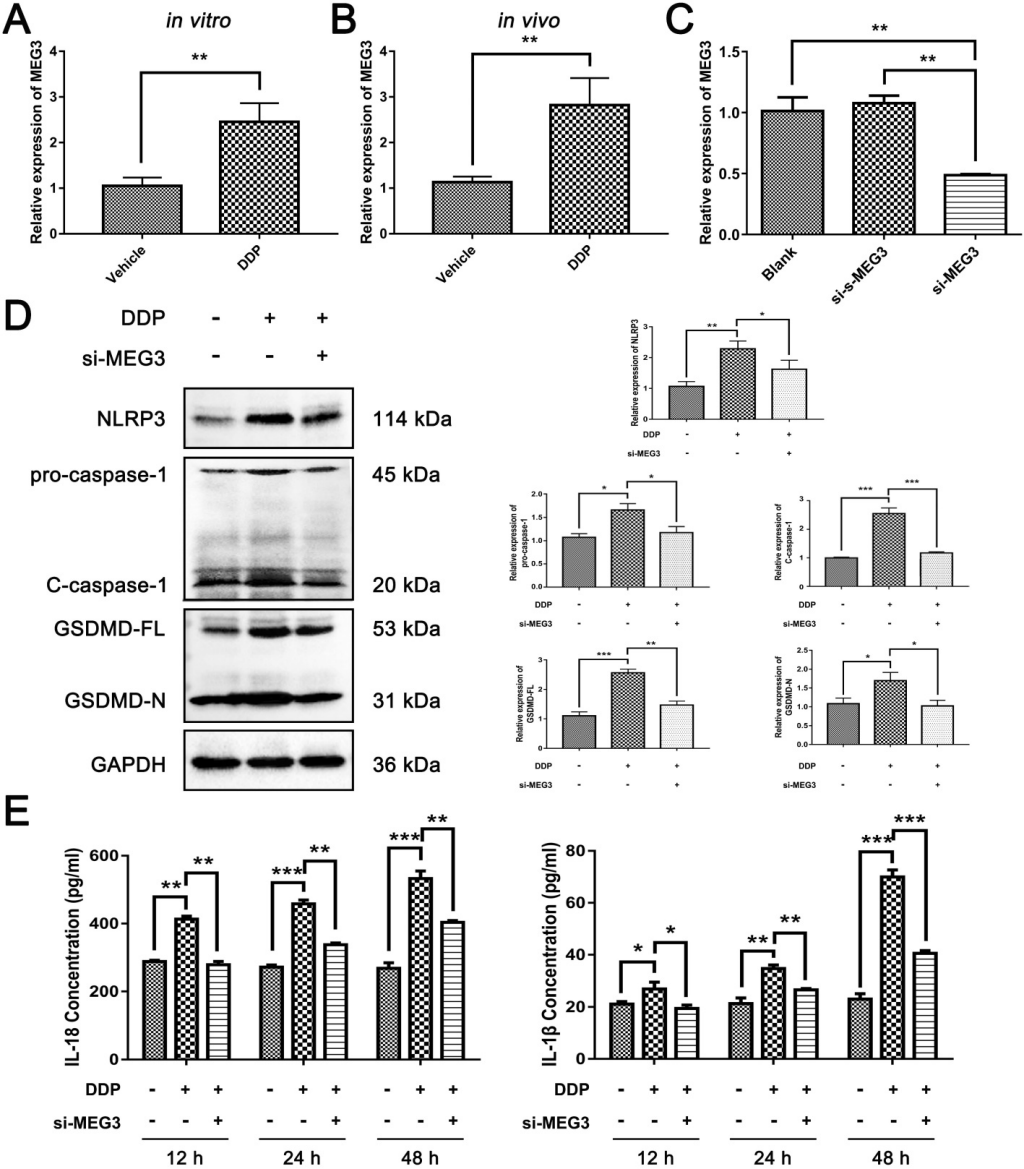
** DDP activated NLRP3/caspase-1/GSDMD pathway by up-regulating MEG3 expression. A and B)** qRT-PCR was performed to detect the expression level of MEG3 in MDA-MB-231 cells (A) and xenograft tumor models (B) response to DDP treatment. **C)** The modulation of MEG3 levels by si-MEG3 was confirmed by qRT-PCR in MDA-MB-231 cells. **D)** Western blot analysis of NLRP3, caspase-1, and GSDMD in different groups with or without DDP treatment for 48 h. GAPDH was included as a loading control. **E)** The levels of IL-18 and IL-1β in the cell culture supernatant of different groups after DDP treatment for the indicated time were determined by ELISA assay. The dose of DDP *in vitro* was 9.952 µM, and the dose of DDP *in vivo* was 5 mg/kg. All data are representative of three independent experiments. Data are shown as mean ± SD. si-MEG3^-^DDP^-^, vehicle treatment group; si-MEG3^-^DDP^+^, si-s-MEG3 + DDP treatment group; si-MEG3^+^DDP^+^, si-MEG3 + DDP treatment group. si-MEG3, siRNA targeting MEG3; si-s-MEG3, negative control of si-MEG3. *P<0.05; **P<0.01; ***P<0.001.

We next continued to explore how DDP regulates NLRP3/caspase-1/GSDMD pathway in MDA-MB-231 cells. We found that MEG3, a kind of lncRNA, was increased significantly in MDA-MB-231 cells exposed to DDP cytotoxicity (Fig. 3A). Consistently, the expression of MEG3 was also remarkably upregulated in the DDP-treated xenograft tumor model *in vivo* (Fig. 3B). To determine the role of MEG3 in the NLRP3/caspase-1/GSDMD pathway activated by DDP, siRNAs targeting MEG3 (si-MEG3) and the negative control of scramble RNA for si-MEG3 (si-s-MEG3) were designed, and the downregulation of MEG3 by si-MEG3 transfection in MDA-MB-231 cells was confirmed by qRT-PCR (Fig. 3C). Notably, the upregulation of NLRP3 induced by DDP was remarkably abolished by si-MEG3 (Fig. 3D). Meanwhile, DDP-activated caspase-1 was also blocked by si-MEG3 as reflected by the decrease of pro-caspase-1 and C-caspase-1 in the si-MEG3 + DDP treatment group (Fig. 3D). Moreover, si-MEG3 significantly downregulated GSDMD-FL and GSDMD-N which were elevated by DDP (Fig. 3D). In addition, the release of IL-18 and IL-1β induced by DDP were also remarkably downregulated in the presence of si-MEG3 and DDP (Fig. 3E). The data indicated that knockdown of MEG3 by si-MEG3 could inhibit the activation of NLRP3/caspase-1/GSDMD pathway by DDP, thus, we speculated that DDP may activate the NLRP3/caspase-1/GSDMD pathway by up-regulating MEG3 expression.

### Knockdown of MEG3 abolishes the activation effect of DDP on pyroptosis

**Figure 4:**
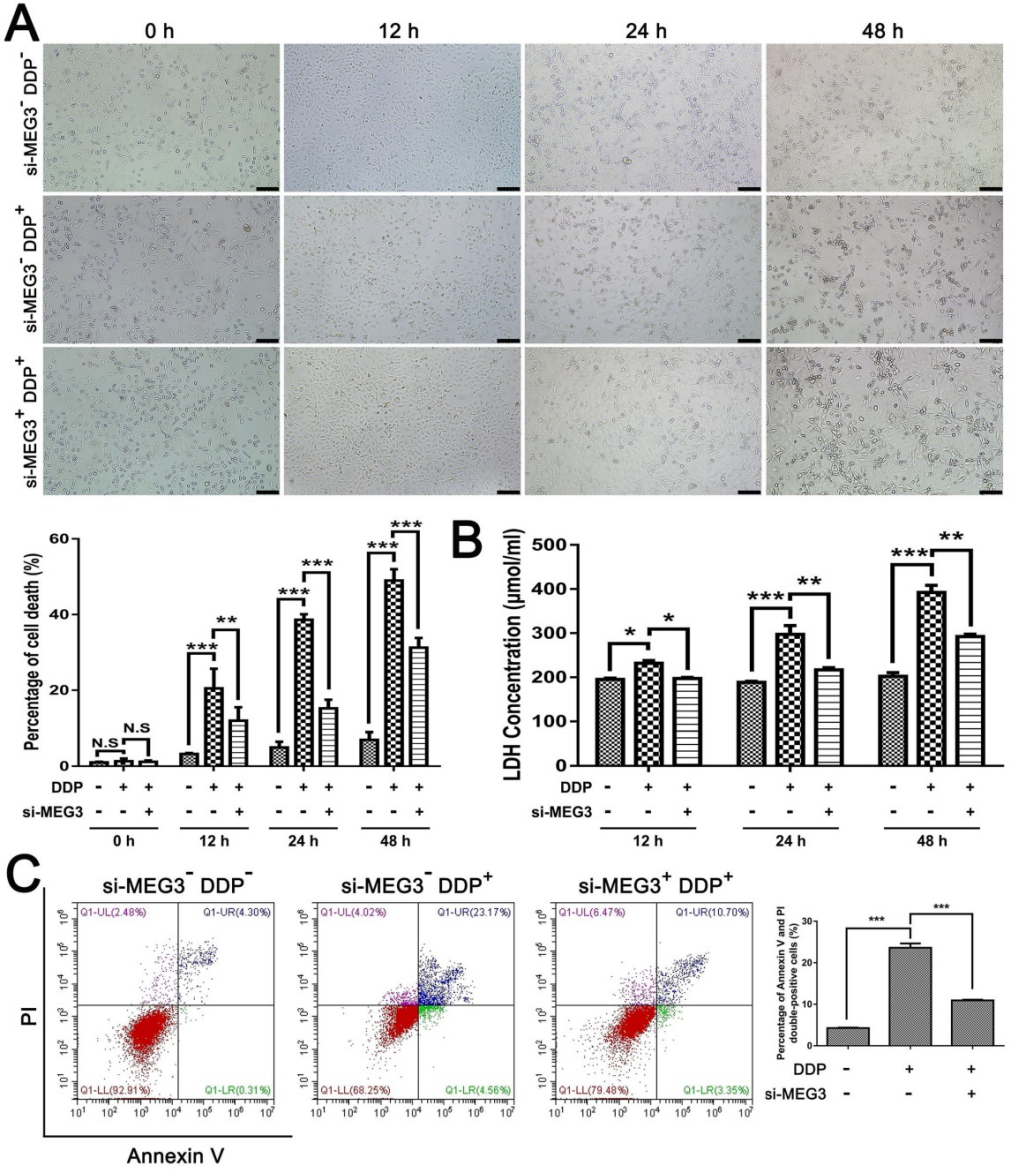
** Knockdown of MEG3 abolished the activation effect of DDP on pyroptosis. A)** Cell morphology images of different groups were taken at the indicated time points when the cells were fixed. Scale bar, 100 µm. The percentage of cell death was quantified according to the cell morphology in 5 randomly chosen fields each containing ~100 cells. **B)** Cytotoxicity of MDA-MB-231 cells in different groups was measured by LDH release in the culture supernatants at the indicated time points. **C)** Flow-cytometry analyses using annexin V and PI staining were performed to test the type of cell death in different groups with or without DDP treatment for 24 h. The dose of DDP in the above-mentioned* in vitro* experiments was 9.952 µM. All data are representative of three independent experiments. Data are shown as mean ± SD. N. S, not significant. si-MEG3^-^DDP^-^, vehicle treatment group; si-MEG3^-^DDP^+^, si-s-MEG3 + DDP treatment group; si-MEG3^+^DDP^+^, si-MEG3 + DDP treatment group. *P<0.05; **P<0. 01, ***P<0.001.

Then, we further investigated the role of MEG3 in DDP-mediated pro-pyroptotic cell death. Time-dependent cell death was observed in MDA-MB-231 cells exposed to DDP cytotoxicity but was consistently rescued by si-MEG3 co-treatment (Fig. 4A), suggesting that knockdown of MEG3 may protect cell death in DDP-treated MDA-MB-231 cells. In addition, we found LDH release in the culture supernatants was increased in a time-dependent manner in the presence of DDP but was inhibited by si-MEG3 + DDP treatment (Fig. 4B), indicating that knockdown of MEG3 may inhibit the damage of cell membrane integrity caused by DDP. Moreover, flow-cytometry analyses showed that the elevated percentage of annexin V and PI double-positive cells by DDP treatment was abolished in the si-MEG3 + DDP treatment group (Fig. 4C), indicating that the activation effect of DDP on pyroptotic cell death was blocked by knockdown of MEG3. Taken together, we speculated that the knockdown of MEG3 abolished the activation effect of DDP on pyroptotic cell death.

### Knockdown of MEG3 blocks the inhibitory effect of DDP on cell proliferation, colony formation, and migration

**Figure 5:**
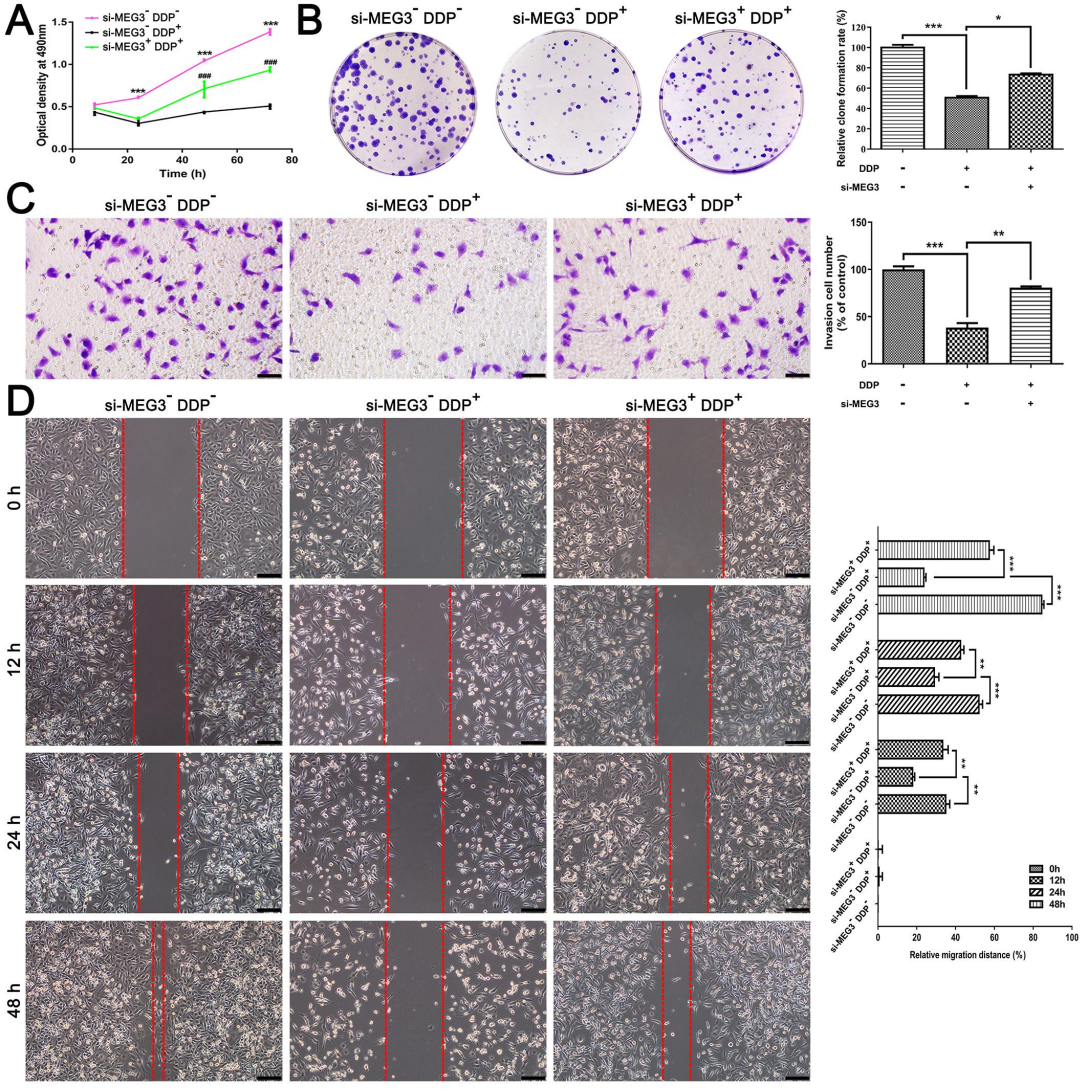
** Knockdown of MEG3 blocked the inhibitory effect of DDP on cell proliferation, colony formation, and migration. A)** Optical density at 490 nm was detected with MTT assay to evaluate the cell viability at the indicated time points. Comparisons were made between the si-MEG3^-^ DDP^-^ treatment group and si-MEG3^-^ DDP^+^ treatment group (***P<0.001), and between the si-MEG3^-^ DDP^+^ treatment group and si-MEG3^+^ DDP^+^ treatment group (^###^*P*<0.001). **B)** Colony formation assay was performed to detect cell proliferation in different groups. **C)** Transwell assay of the migration and invasion abilities in different groups. Scale bar, 50 µm. **D)** Analysis of migration by means of wound healing. Images of the wound area were taken at four different time points (0, 12, 24, and 48 h). Scale bar, 100 µm. The dose of DDP in the above-mentioned *in vitro* experiments was 9.952 µM. All data are representative of three independent experiments. Data are shown as mean ± SD. si-MEG3^-^DDP^-^, vehicle treatment group; si-MEG3^-^DDP^+^, si-s-MEG3 + DDP treatment group; si-MEG3^+^DDP^+^, si-MEG3 + DDP treatment group. *P<0.05; **P<0.01; ***P<0.001; ^###^*P*<0.001.

Based on the above results, DDP may induce pyroptosis via activation of the MEG3/NLRP3/caspase-1/GSDMD pathway in TNBC. To further validate the role of pyroptosis on DDP chemotherapy sensitivity, we examined cell proliferation, colony formation, and migration* in vitro*. MTT assay showed that cell proliferation was significantly inhibited in a time-dependent manner of MDA-MB-231 cells responding to DDP treatment (Fig. 5A). However, the cell viability was remarkably recovered when cells treated with si-MEG3 + DDP after 48h (such as 48 h and 72 h shown in Fig. 5A), suggesting that knockdown of MEG3 blocked the inhibitory effect of DDP on cell proliferation. Besides, colony formation assay indicated that the long-term clonogenic survival of MDA-MB-231 cells was remarkably diminished by DDP, while was reversed by si-MEG3 (Fig. 5B). Moreover, transwell assay and wound healing test demonstrated that DDP suppressed the migration ability of MDA-MB-231 cells (Fig. 5C and 5D), while the migration ability was substantially enhanced by si-MEG3, as reflected by the increased number of migrated cells (Fig. 5C) and decreased wound area (Fig. 5D). Accordingly, the knockdown of MEG3 blocked the inhibitory effect of DDP on cell proliferation, colony formation, and migration *in vitro*.

### DDP-induced pyroptosis suppresses tumor growth and metastasis via MEG3/NLRP3/caspase-1/GSDMD pathway in a xenograft animal model

**Figure 6:**
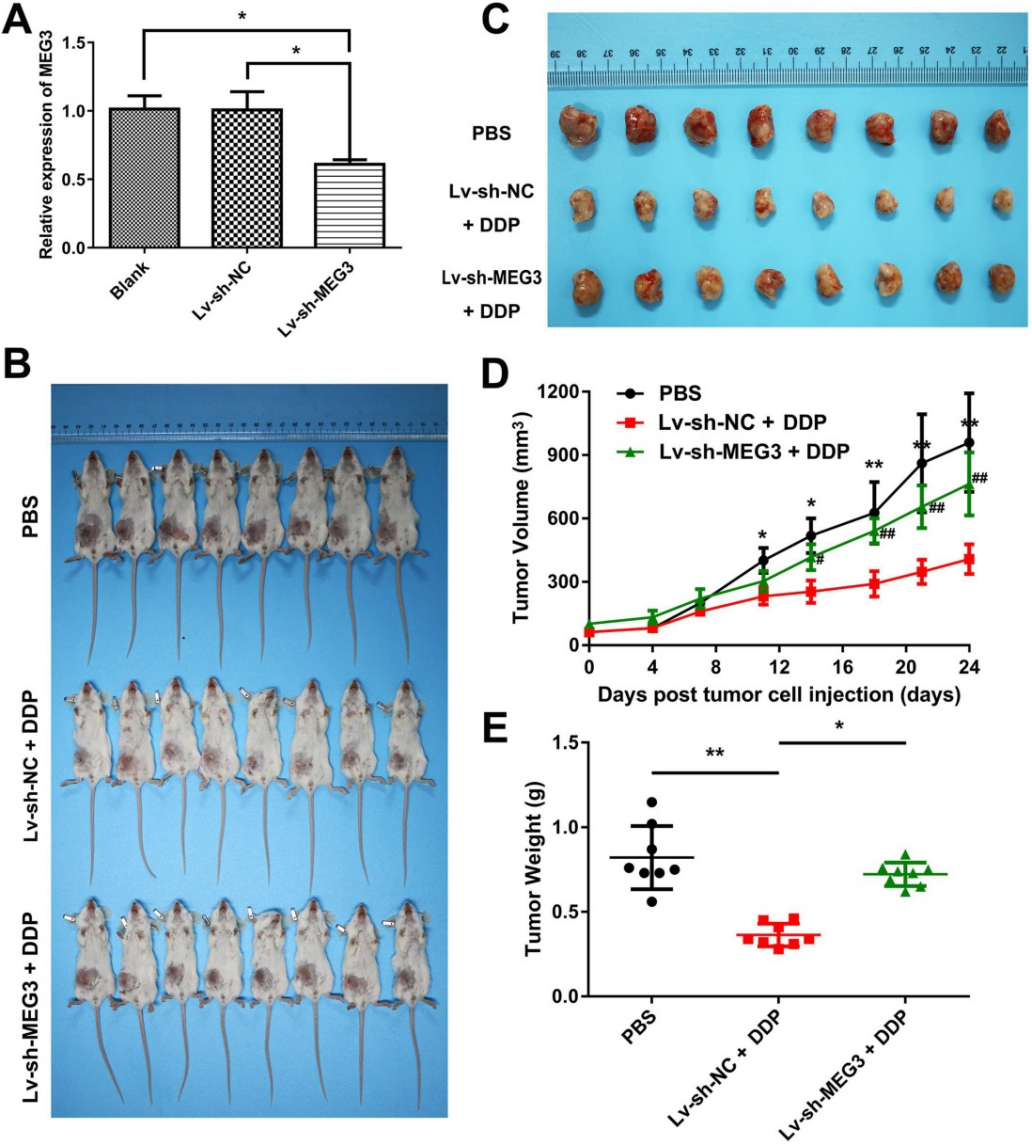
** Knockdown of MEG3 promoted tumor growth and inhibited DDP chemotherapy sensitivity *in vivo*. A)** Effect of MEG3 knockdown was confirmed by qRT-PCR. *P<0.05. **B)** Tumor sizes in BALB/C nude mice of three treatment groups. **C)** Representative images of tumors taken at the end of the study. **D)** Tumor growth curves of three treatment groups. Comparisons were made between the PBS negative control group and Lv-sh-NC + DDP treatment group (*P<0.05; **P<0.01), and between the Lv-sh-NC + DDP treatment group and Lv-sh-MEG3 + DDP treatment group (^#^*P*<0.05; ^##^*P*<0.01). Day 0 was defined as the day before PBS or DDP injection. **E)** Tumor weight of three treatment groups. The dose of DDP in the above-mentioned *in vivo* experiments was 5 mg/kg twice a week and continued to 24 days. *P<0.05; **P<0.01. Data are shown as mean ± SD (n=8). Lv-sh-MEG3, recombinant lentivirus expressing shRNA targeting MEG3; Lv-sh-NC, negative control of Lv-sh-MEG3.

To determine whether DDP-induced pyroptosis suppresses tumor growth and metastasis via MEG3/NLRP3/caspase-1/GSDMD pathway *in vivo*, we established a xenograft model of nude mice bearing the MDA-MB-231 cells. A stable MEG3 knockdown cell line was constructed by infecting MDA-MB-231 cells with the recombinant lentivirus expressing short-hairpin RNA (shRNA) targeting MEG3 (Lv-sh-MEG3). A stable expressing Lv-sh-NC cell line (Lv-sh-NC) was considered as the negative control of Lv-sh-MEG3. The effect of knockdown by Lv-sh-MEG3 was confirmed by qRT-PCR (Fig. 6A). Then, the stable MEG3 knockdown cell line (Lv-sh-MEG3), the stable expressing Lv-sh-NC cell line (Lv-sh-NC), or MDA-MB-231 cell line was transplanted into the right thoracic mammary fat pads of immunodeficient BALB/C nude mice to generate xenograft mice. Three treatment groups of xenograft mice were established: PBS treatment group (MDA-MB-231 xenograft mice treated with PBS as negative control), Lv-sh-NC + DDP treatment group (Lv-sh-NC xenograft mice treated with DDP), Lv-sh-MEG3 + DDP treatment group (Lv-sh-MEG3 xenograft mice treated with DDP) (Fig. 6B). Then, tumors were stripped and presented as in Fig. 6C. DDP treatment significantly suppressed the growth of tumor cells when compared to the PBS negative control group (Fig. 6D). However, the growth of the tumors in the Lv-sh-MEG3 + DDP treatment group was significantly faster than that of the tumors in the Lv-sh-NC + DDP treatment group (Fig. 6D). Consistently, tumor weight in the mice of the Lv-sh-MEG3 + DDP treatment group was remarkably greater than that of the Lv-sh-NC + DDP treatment group (Fig. 6E), suggesting that MEG3 knockdown promoted tumor growth and inhibited DDP chemotherapy sensitivity *in vivo*.

**Figure 7:**
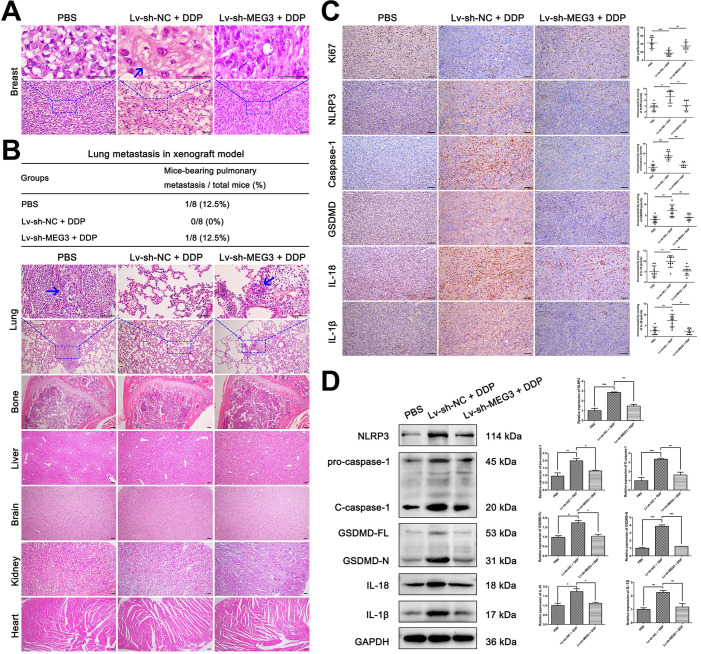
** Knockdown of MEG3 reversed the suppression of DDP on tumor metastasis ability and abolished the activation of NLRP3/caspase-1/GSDMD pathway. A)** H&E staining of breast tumor specimens in xenograft mice. Blue arrows indicated the tumor cells with pyroptosis-like changes. **B)** H&E staining of important organs including lung, bone, liver, brain, kidney, and heart. The blue arrow showed the metastatic tumor cells. The three-line table showed the lung metastasis incidence in the xenograft model. **C)** Immunohistochemical staining of Ki67, NLRP3, caspase-1, GSDMD, IL-18, and IL-1β expression in breast tumor specimens of xenograft mice. **D)** Western blot analysis of NLRP3, caspase-1, GSDMD, IL-18, and IL-1β in breast tumor specimens of xenograft mice. GAPDH was included as a loading control. The dose of DDP in the above-mentioned *in vivo* experiments was 5 mg/kg twice a week and continued to 24 days. Data are shown as mean ± SD (n=8). *P<0.05; **P<0.01; ***P<0.001. Scale bar, 50 µm.

In addition, H&E staining of tumors and several organs were performed to detect the pathological changes of tumor cells and tumor metastasis in xenograft mice. As shown in Fig. 7A, pyroptosis-like changes were observed in the Lv-sh-NC + DDP treatment group (blue arrows), but this phenomenon rarely occurred in the other two groups. Besides, lung metastasis occurred in the PBS negative control group and the Lv-sh-MEG3 + DDP treatment group (Fig. 7B, lung metastasis incidence was 12.5% in both groups), while no significant metastasis was found in the Lv-sh-NC + DDP treatment group. Meanwhile, no significant metastasis was found in other organs including bone, liver, brain, kidney, and heart in all groups (Fig. 7B). Immunohistochemistry (IHC) was also performed to verify whether MEG3 knockdown affected NLRP3/caspase-1/GSDMD pathway *in vivo*. As shown in Fig. 7C, the decrease of proliferation rate (Ki67) and the increase of pyroptosis-related proteins (NLRP3, caspase-1, GSDMD, IL-18, and IL-1β) induced by DDP was abolished by MEG3 knockdown in the Lv-sh-MEG3 + DDP treatment group. Western blot also showed that MEG3 knockdown reversed the upregulation of NLRP3, pro-caspase-1, C-caspase-1, GSDMD-FL, GSDMD-N, IL-18, and IL-1β protein levels induced by DDP (Fig. 7D), further validating that knockdown of MEG3 abolished the activation of NLRP3/caspase-1/GSDMD pathway *in vivo.*

Taken together, these data suggested that DDP-induced pyroptosis suppressed tumor growth and metastasis via MEG3/NLRP3/caspase-1/GSDMD pathway in a xenograft animal model.

## Discussion

**Figure 8:**
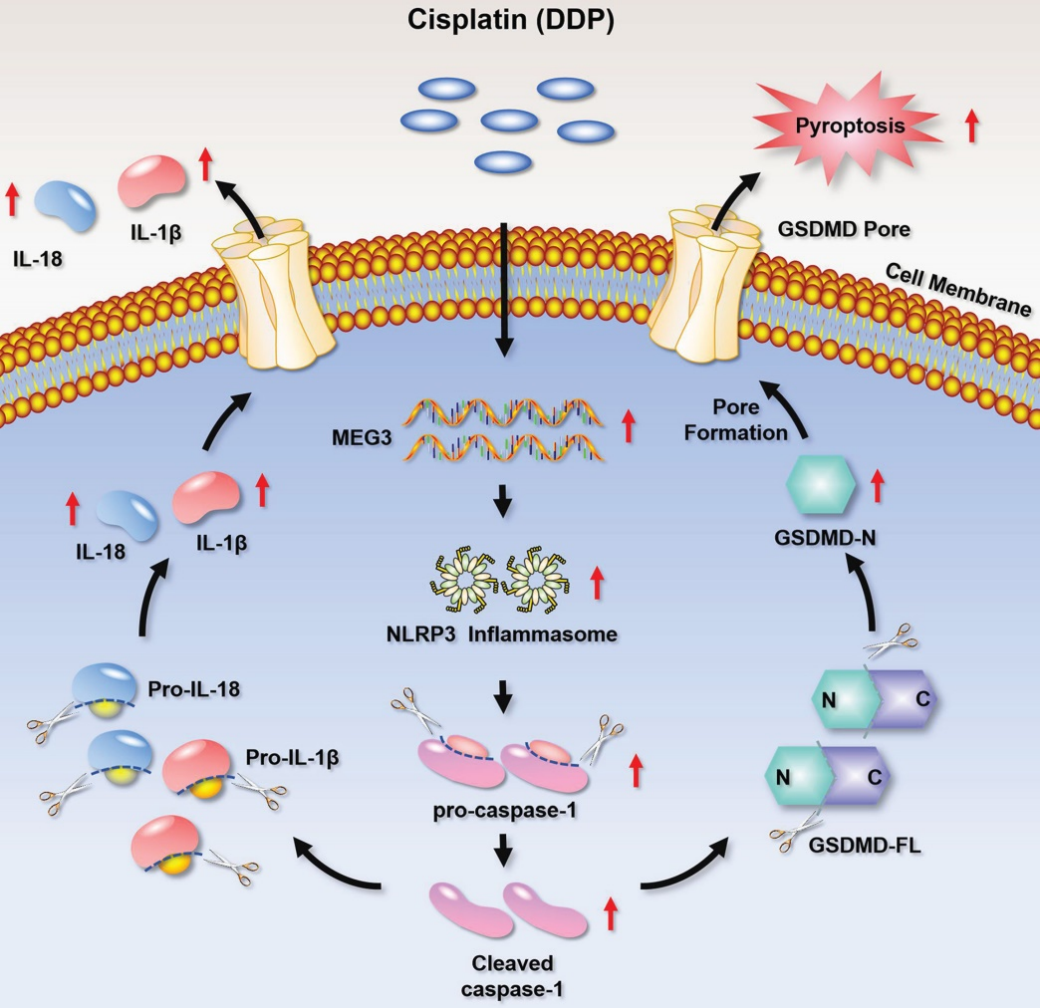
A schematic diagram representing that DDP induces pyroptosis in TNBC via activation of MEG3/NLRP3/caspase-1/GSDMD pathway.

In the current study, we found that DDP-based NACT induced pyroptosis-like changes in TNBC tissues and up-regulated pyroptosis-related proteins. Further mechanism studies clarified that the chemotherapy drug DDP promoted the activation of NLRP3 inflammasome by up-regulating MEG3, and further activated the caspase-1-dependent pyroptosis pathway. Activated caspase-1 cleaved GSDMD and released GSDMD N-terminal fragment, thereby forming the membrane pore. On the other hand, caspase-1 activation promoted the maturation and secretion of the cytokines IL-18 and IL-1β precursors, and resulted in a release of IL-18 and IL-1β from the membrane pores. Therefore, the MEG3/NLRP3/caspase-1/GSDMD pathway may be one of the important mechanisms for DDP to induce pyroptosis and exert anti-tumor effects in TNBC (Fig. 8). We demonstrated for the first time that DDP induces pyroptosis via activation of the MEG3/NLRP3/caspase-1/GSDMD pathway in TNBC.

A growing body of preclinical and clinical data suggests that platinum chemotherapy has a potential role in the treatment of TNBC 5, 6, 28. However, the mechanism underlying these effects has not been fully elucidated. Here, in resected TNBC specimens post DDP-based NACT, tumor cells displayed cell swelling, unclear cell membrane boundary, and increased inflammation, which were similar to several characteristics of pyroptosis. Pyroptosis is a novel manner of cell death that can be mediated by chemotherapy drugs and is regarded as a new direction of anti-tumor research. Emerging evidence has shown that DDP could induce pyroptotic cell death in various cells 9, 29. Enhancing the pyroptotic effect can increase the chemosensitivity to DDP in several cancer cells, including esophageal cancer cells 30, oesophageal squamous cell carcinoma cells 31, gastric cancer cells 29, and non-small cell lung cancer cells 32. Nevertheless, it is still unclear whether DDP induces TNBC cell pyroptosis. In this study, we reported for the first time that DDP could induce pyroptosis in TNBC cells* in vitro* and *in vivo*. Some of the dying MDA-MB-231 cells responding to DDP treatment displayed characteristic morphology of pyroptosis, as reflected by large bubbles blowing from the cellular membrane accompanied by increased Annexin V-PI double staining and LDH release. Additionally, pyroptosis-like changes were also observed in DDP-treated xenograft mice, which further confirmed that DDP could induce pyroptosis in TNBC.

In recent years, multiple studies were focusing on the in-depth understanding of the underlying regulatory mechanism of pyroptosis. Membrane rupture is the final event of pyroptosis cell death. GSDMD and GSDME are two kinds of pore-forming proteins that are extensively studied in pyroptosis 9, 17. GSDME-mediated pyroptosis has recently been reported to be triggered by activated caspase-3 to form the N-terminal fragment of GSDME (GSDME-N), which results in cell membrane pore formation 9. Most studies indicated that the GSDME-mediated pyroptosis was induced in the chemotherapeutic agent-treated cancer cells as well as normal cells 9-11, 31, 33. While GSDMD-mediated pyroptosis was usually reported in immune cells upon inflammatory stimulations 17, 34, 35, and only a few studies reported that it occurred in the chemotherapeutic agent-treated cancer cells 32. However, we found that the GSDMD N-terminal fragment was induced in DDP-treated MDA-MB-231 cells, which suggested that GSDMD was cleaved exposed to DDP cytotoxicity. Moreover, the activation of NLRP3 inflammasome was also observed responding to DDP treatment *in vitro* and *in vivo*, as reflected by the elevated NLRP3, pro-caspase-1, and C-caspase-1, and the accumulation of IL-18 and IL-1β in DDP-treated MDA-MB-231 cells and xenograft mice. Taken together, these data provided direct evidence that DDP could induce pyroptosis via activation of the NLRP3/caspase-1/GSDMD pyroptosis pathway in TNBC.

Although the role of NLRP3 inflammasome in pyroptosis has been described, its upstream regulatory mechanism is less well characterized. Recently, a mounting body of evidence uncovered that lncRNAs may contribute to NLRP3 inflammasome activation 24, 36, 37. MEG3 is a type of lncRNA that has been studied extensively in tumor research. At present, most studies focused on its inhibitory effect on cell proliferation, but little is known about its role in cell pyroptosis. Emerging evidence showed that melatonin prevented endothelial cell pyroptosis via regulation of the MEG3/miR-223/NLRP3 axis, suggesting that MEG3 may be one of the regulatory factors of NLRP3 inflammasome activation 24. In addition, another study on the pathogenesis of ischemic stroke indicated that MEG3 promoted cerebral ischemia-reperfusion injury through increasing pyroptosis by targeting miR-485/AIM2 axis 26. A recent study also showed that knockdown of MEG3 alleviated hyperoxia-induced lung injury via inhibiting pyroptosis 38. These studies suggested that MEG3 may be involved in pyroptosis initiation and development in different diseases. However, the biological role of the MEG3/NLRP3 axis in DDP-induced pyroptosis of TNBC cells has not been reported. In this study, we found that DDP significantly upregulated MEG3 expression *in vitro* and *in vivo*. In addition, the upregulation of NLRP3, pro-caspase-1, C-caspase-1, GSDMD-FL, GSDMD-N, IL-18, and IL-1β induced by DDP were remarkably abolished by knockdown of MEG3 *in vitro* and* in vivo*, further confirming that DDP activated NLRP3/caspase-1/GSDMD pathway by up-regulating MEG3 expression. Furthermore, knockdown of MEG3 prevented the increase of the pyroptotic cell percentage, LDH release as well as the time-dependent cell death caused by DDP, which suggested that knockdown of MEG3 abolished the activation effect of DDP on pyroptosis.

Finally, in order to extend the clinical significance of MEG3-mediated pyroptosis in the DDP-induced anti-tumor effect of TNBC, we examined the tumor growth and metastasis ability* in vitro* and* in vivo*. We found that the cell proliferation, long-term clonogenic survival ability, and migration ability were remarkably suppressed by DDP, while reversed by knockdown of MEG3, suggesting that knockdown of MEG3 blocked the inhibitory effect of DDP on cell proliferation, colony formation, and migration. Furthermore, DDP treatment significantly suppressed the growth of tumor cells and metastasis ability in xenograft animal models, otherwise, MEG3 knockdown reversed the inhibition of tumor growth and metastasis ability caused by DDP, which provided evidence that MEG3 knockdown could inhibit DDP chemotherapy sensitivity *in vivo*.

In conclusion, we demonstrated for the first time that DDP induced pyroptosis via activation of MEG3/NLRP3/caspase-1/GSDMD pathway in TNBC *in vitro* and *in vivo.* Our findings uncovered a novel mechanism of DDP-induced pyroptosis in TNBC, which may help to develop new strategies for the therapeutic interventions in TNBC. Although the current results expand our understanding of the mechanism of pyroptosis induced by DDP in TNBC, there are still some limitations in this study. Since GSDMD and GSDME are two important pyroptotic substrates, we only examined the role of GSDMD-mediated pyroptosis in TNBC. Thus, further research is needed to investigate whether GSDME-dependent pyroptosis is involved in the DDP-induced anti-tumor effect of TNBC. On the other hand, long-term chemotherapeutic drug stimulation will render TNBC patients resistant, how to improve the sensitivity to chemotherapy is a major problem in clinical treatment. In the present study, we found that knockdown of MEG3 inhibited DDP chemotherapy sensitivity in TNBC. However, whether up-regulation of MEG3 expression could enhance the chemosensitivity of DDP-resistant TNBC cells have not been clarified. Therefore, DDP-resistant cell lines should be constructed to detect the effect of overexpression of MEG3 on the chemosensitivity of DDP in future research. In addition, another limitation of the present study is that we only used a single breast cancer cell line to investigate the potential roles of silencing MEG3 on pyroptosis, it is difficult to confirm whether this effect is universal across other TNBC cell lines. And, strictly speaking, as knockdown of MEG3 only partly abolished the activation of DDP-induced NLRP3/caspase-1/GSDMD pathway and anti-cancer functions in MDA-MB-231 cells, MEG3 may partially mediated the pyroptotic signaling upon DDP treatment in this type of TNBC cell line. To further validate these fundamental *in vitro* and *in vivo* results, we will involve the use of other types of TNBC cell models (such as MDA-MB-468, 4T1, and patient-derived xenograft cell models) in future studies.

## Abbreviations

DDP: cisplatin
TNBC: triple-negative breast cancer
lncRNA: long non-coding RNA
MEG3: maternally expressed gene 3
ER: estrogen receptor
PR: progesterone receptor
HER2: human epidermal growth factor receptor 2
NACT: Neoadjuvant chemotherapy
pCR: pathological complete response
PCD: programmed cell death
NLRP3: NOD-like receptor family pyrin domain-containing 3
GSDMD: gasdermin D
GSDMD-N: GSDMD N-terminal fragment
DMEM: Dulbecco's modified Eagle's minimal essential medium
FBS: fetal bovine serum
si-MEG3: siRNAs targeting MEG3
si-s-MEG3: scramble RNA for si-MEG3
SD: standard deviation
IC50: median inhibitory concentration
MTT: 3-(4,5-dimethyl-2-thiazolyl)-2, 5-diphenyltetrazolium bromide
PI: propidium iodide
LDH: lactate dehydrogenase
GSDMD-FL: full-length GSDMD
C-caspase-1: cleavage of caspase-1
qRT-PCR: quantitative reverse transcription polymerase chain reaction
shRNA: short-hairpin RNA
Lv-sh-MEG3: lentivirus expressing shRNA targeting MEG3
Lv-sh-NC: negative control of Lv-sh-MEG3
IHC: immunohistochemistry
GSDME-N: N-terminal fragment of GSDME
